# Metabolic Profiling of *Candida auris*, a Newly-Emerging Multi-Drug Resistant *Candida* Species, by GC-MS

**DOI:** 10.3390/molecules24030399

**Published:** 2019-01-22

**Authors:** Mohammad H. Semreen, Sameh S. M. Soliman, Balsam Q. Saeed, Abdullah Alqarihi, Priya Uppuluri, Ashraf S. Ibrahim

**Affiliations:** 1 Sharjah Institute for Medical Research and College of Pharmacy, University of Sharjah, P.O. Box 27272, Sharjah, UAE; msemreen@sharjah.ac.ae; 2 Department of Clinical Sciences, College of Medicine, University of Sharjah, P.O. Box 27272, Sharjah, UAE; bsaeed@sharjah.ac.ae; 3 Division of Infectious Diseases, Los Angeles Biomedical Research Institute, Harbor-UCLA Medical Center, Torrance, CA 90509, USA; aalqarihi@labiomed.org (A.A.); puppuluri@labiomed.org (P.U.); ibrahim@labiomed.org (A.S.I.); 4 David Geffen School of Medicine at UCLA, Los Angeles, CA 90095, USA

**Keywords:** *Candida auris*, metabolic profiling, GC-MS, morphogenesis, hyphae, yeast

## Abstract

*Candida auris*, a newly-emerging *Candida* species, is a serious global health threat due to its multi-drug resistant pattern, difficulty to diagnose, and the high mortality associated with its invasive and bloodstream infections. Unlike *C. albicans*, and *C. dubliniensis* which can form true hyphae, *C. auris* grows as yeast or pseudohyphae and is capable of developing biofilms. The reasons for the inability of *C. auris* to form true hyphae are currently unknown. Metabolites secreted by microorganisms, including *Candida*, are known as important factors in controlling morphogenesis and pathogenesis. Metabolic profiling of *C. auris* and *C. albicans* cultures was performed using gas chromatography–mass spectrometry (GC–MS). Compared to *C. albicans*, *C. auris* secreted several hyphae-inhibiting metabolites, including phenylethyl, benzyl and isoamyl alcohols. Furthermore, a biofilm-forming metabolite—tyrosol—was identified. On the other hand, several other biomarkers identified from *C. auris* but not from *C. albicans* cultures may be produced by the organism to overcome the host immune system or control fungal adaptations, and hence ease its invasion and infections. The results from this study are considered as the first identification of *C. auris* metabolic activities as a step forward to understand its virulence mechanisms.

## 1. Introduction

*Candida* species, in particular *C. albicans*, are responsible for most fungal infections in humans. Although *Candida* species can cause superficial mucosal and skin infections, they can generate serious threats to immunocompromised patients, including fatal bloodstream candidiasis [1]. In recent years, the Centers for Disease Control and Prevention (CDC) has designated *C. auris* as an emerging serious global health threat mainly because of its multidrug-resistant pattern associated with high mortality rate that can reach 60% [2,3], difficulty to diagnose [4] and recent outbreaks in healthcare settings owing to the yeast persistence as a colonizer on abiotic surfaces [5].

*C. auris* was first identified in Japan on 2009 [6]. Since then, infections due to *C. auris* have been reported from over a dozen countries, including United States, Canada, Colombia, Germany, India, Israel, Japan, Kenya, Norway, Pakistan, Spain, South Africa, South Korea, the United Kingdom, Venezuela, Kuwait and Oman [7]. Phylogenetic analysis indicates that *C. auris* is a recent and simultaneous emergent strain in different geographical areas [8]. This yeast can be recovered from several human specimens, including sterile body fluids, ears, wounds and mucocutaneous swabs. However, the major manifestation of the infection includes invasive and bloodstream infections [9]. Like other *Candida* species, there is no indication that it can cause true respiratory, urinary, and skin and soft tissue infections despite being isolated from such samples [10,11]. Individuals who are at risk of acquiring *C. auris* infections include hospitalized and nursing home patients. These patients usually have comorbidities that is likely to contribute with the multi-drug resistant nature of the yeast to the lethality of the infection [12]. Importantly, *C. auris* is often misdiagnosed as *C. haemulonii*. Thus, the CDC recommends further testing whenever a diagnosis of *C. haemulonii* is identified [4]. As *C. auris* continues to emerge throughout the world, vital measures are required to slow down its spread and to reduce the environmental contamination in healthcare facilities and to improve diagnosis of the infection.

*C*. *auris* exhibits round-to-ovoid yeast morphology and, in some reports, ability to form pseudohyphae without the formation of true hyphae [12]. A major strategy used by *Candida albicans* to control its morphogenesis is the production of phenotype-switching metabolites [13]. The concentration of these metabolites increases to a critical threshold, which in turn triggers a morphogenetic regulatory response [14]. Several *C. albicans* metabolites, including farnesol, are known to inhibit yeast-to-hyphae formation [15]. In contrast, *C. albicans* tyrosol induces filamentation and hence biofilm formation [16]. Other reported hyphae-inhibiting alcohols include phenylethyl [17], isoamyl and benzyl alcohols in addition to tryptophol [18].

We conducted GC-MS analysis to compare the metabolic profile of *C. auris* to those of *C. albicans*. GC-MS is a powerful technique used to establish a comprehensive quantitative metabolic profiling of volatile small molecules from any complex biological samples [19]. The results showed that *C. auris* produces metabolites consistent with those promoting yeast formation.

## 2. Results and Discussion

*C. auris* or *C. albicans* cultures were extracted and derivatized prior to GC-MS analysis in order to enhance the appearance of metabolic spectrum and increase the detection limits of the metabolites. The identified metabolites from *Candida* cultures were classified in relation to their possible morphogenic and virulence activities.

### 2.1. Superior Production of Hyphae-Inhibitory Metabolites by C. auris

It is reported that, *C. albicans* responds to environmental changes by developing different polymorphic forms including yeast, pseudo-hyphae and true hyphae [20,21]. Phenotype-switching metabolites play a critical role in *C. albicans* morphogenesis [22]. Unlike *C. albicans, C. auris* grows readily as yeast form and does not produce true hyphae [3,12,23]. Further reports indicated that *C. auris* is capable of forming biofilms [24] and adhering to catheter material [25].

**Figure 1:**
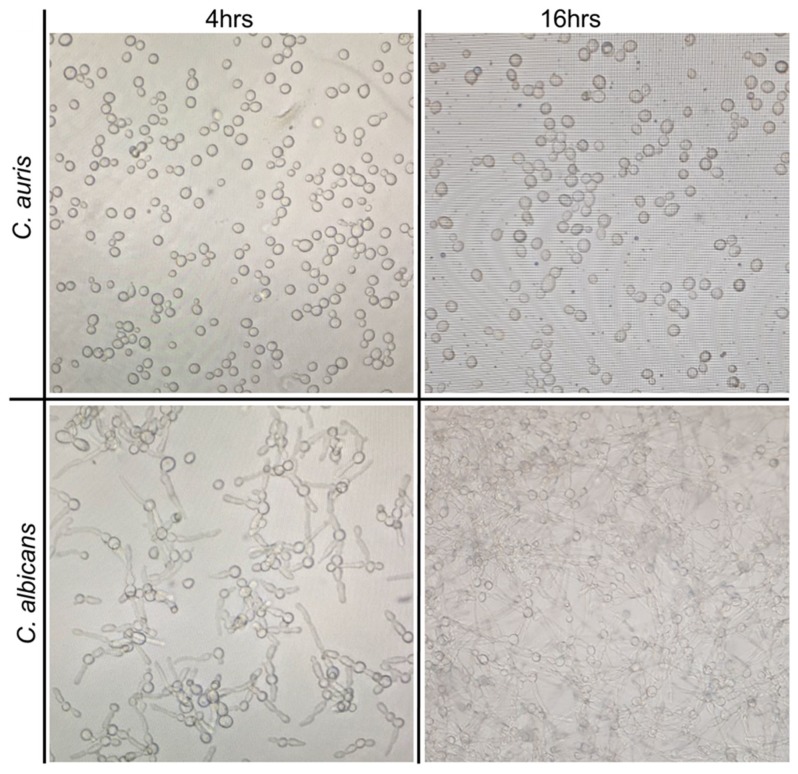
Growth of *C. auris* (CAU09) in comparison to *C. albicans* at biofilm-forming condition at 4 and 16 h. *Candida* isolates were cultured at 37 °C in 125 mL Corning culture flasks.

**Figure 2:**
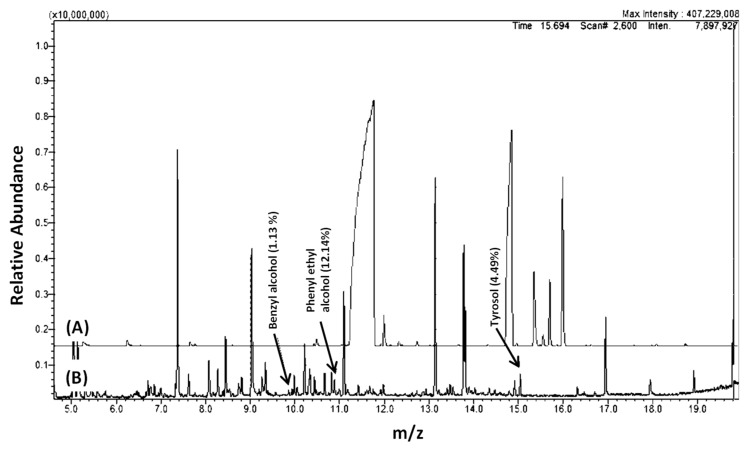

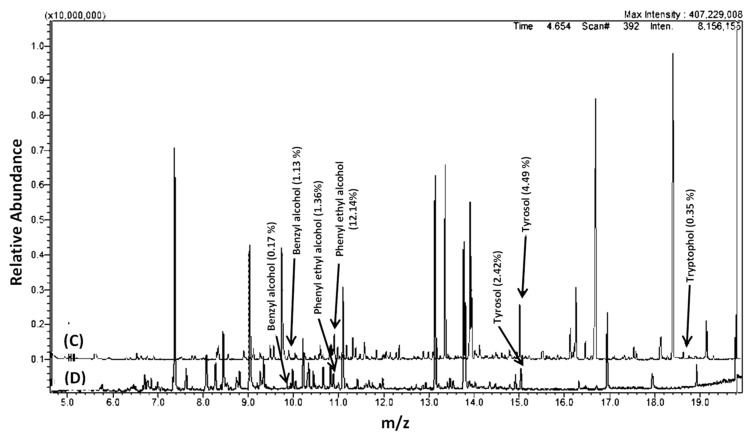
GC-MS chromatograms of *Candida* cultures extracts. Chromatograms of (**A**) *C. albicans* ATCC10231 strain compared to (**B**) *C. auris* (CAU09) strain. (**C**) and (**D**) GC-MS chromatograms of *C. auris* CAU09 at two different growth stages (**C**) 16 h compared to (**D**) 4 h incubation periods representing the differences in metabolites abundance. CAU09 was used as a representative to *C. auris* strains since all *C. auris* strains showed a similar pattern of GC-MS analysis (data not shown). Cell-free supernatants were collected, extracted by chloroform and derivatized by MSTFA and TMS prior to GC-MS analysis using GC-2010 coupled with a GC–MS QP-2010 Ultra.

**Figure 3:**
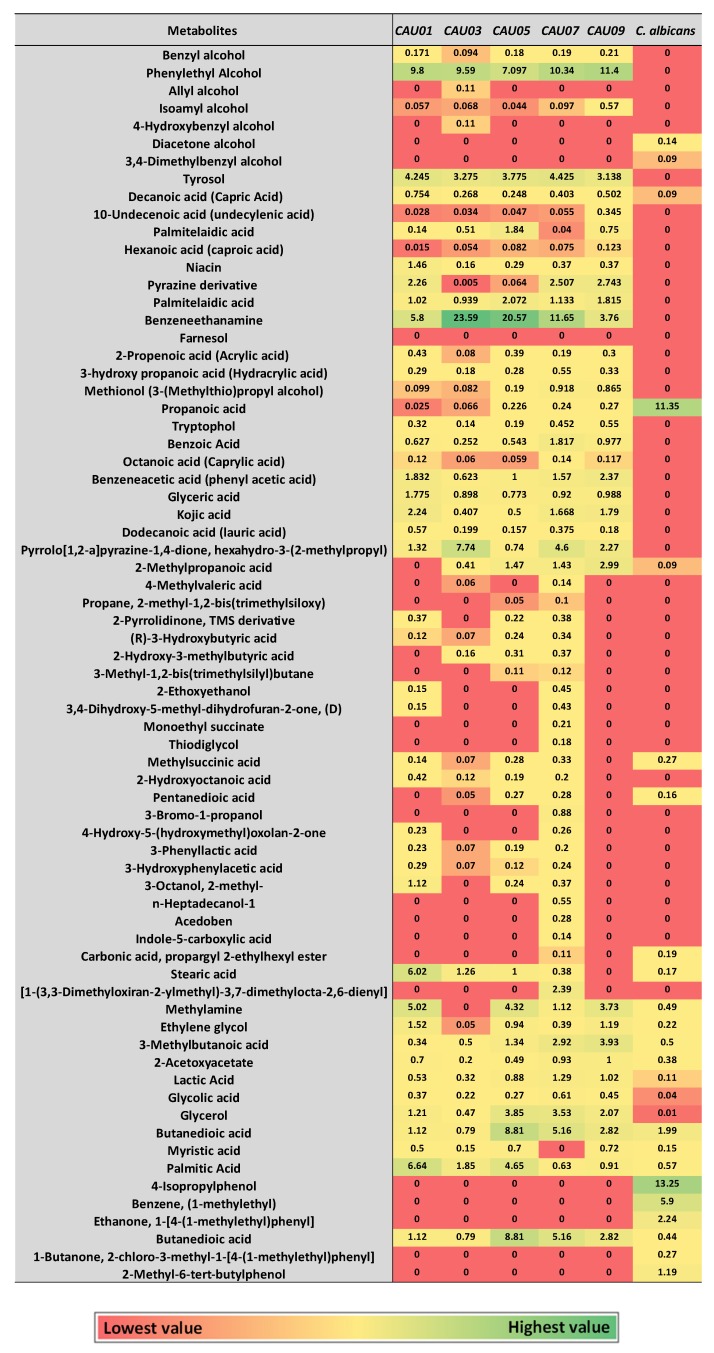
Heatmap of major identified metabolites in *Candida* cultures by GC-MS. Metabolite average relative percentage of four experimental replicates from two independent biological experiments were displayed as colors ranging from red to green as shown in the key. The relative percentage of a metabolite represented in relation to total areas of all detected metabolites in an extract.

We wanted to investigate if metabolites production by *C. auris* is consistent with its yeast form, even when grown under hyphae-promoting conditions. Thus a preliminary study was conducted by growing *C. auris* in culture conditions that mimic the development of hyphae in *C. albicans* (Figure 1). Cell free supernatants from cultures of *C. auris* CAU09 and *C. albicans* were then collected and separately screened for the presence of similar metabolites using GC-MS analysis (Figure 2A–D). The results showed that *C. auris* CAU09 cultures secreted metabolites particularly: 1) aromatic alcohols such as phenylethyl alcohol, benzyl alcohol, isoamyl alcohol and tyrosol; and 2) acids such as benzoic, benzenacetic, glyceric and others that were not identified in *C. albicans* cultures (Figure 2A,B and Figure 3).

**Figure 4:**
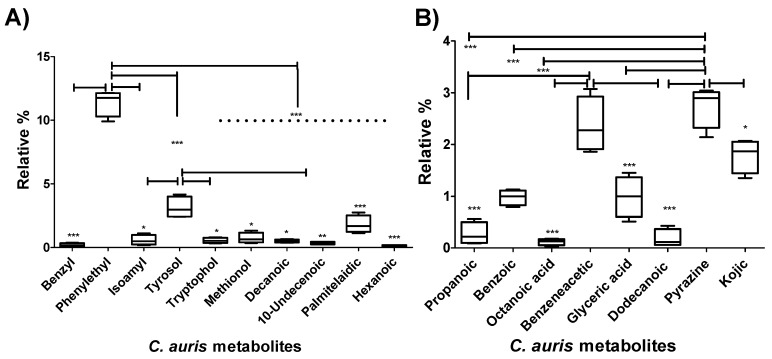
Relative percentages of metabolites produced by *C. auris* CAU09 cultures. (**A**) The metabolites produced by the organism for the purpose of its morphogenic changes. (**B**) The metabolites produced by the organism for the purpose of protection and colonization. The quantity of each metabolite was represented as the relative percentage by measuring the area under the peak of each metabolite in relation to the total areas of all other metabolites detected in the extract. The data display the mean of the relative percentage ± standard error of the mean. The data was graphed using Box-and-Whiskers Plots and analyzed by one-way analysis of variance (ANOVA) using Bonferroni’s Multiple Comparison Test. *P* value < 0.05 was considered as significant. The standard error represents the mean of 4 replicas of two independent experiments. The level of significance was indicated by asterisks.

Metabolites known to be involved in *Candida* morphogenic changes were grouped and their average relative percentages were compared to each other in *C. auris* CAU09 strain using Box-and-Whiskers Plots (Figure 4A). The plots showed that there was a significant increase in the production of both phenylethyl alcohol (11.39 ± 0.5%) and tyrosol (3.137 ± 0.4). In addition, a less prominent increase in palmitelaidic acid (1.8 ± 0.34), and at lower extent, benzyl alcohol (0.2 ± 0.06%) as well as isoamyl alcohol (0.57 ± 0.19%) was detected (Figure 4A and Supplementary Table S1).

The secreted metabolites in CAU09 cultures were further compared at 4 and 16 h incubation periods (Figure 2C,D). Both phenylethyl alcohol, and tyrosol, were increased by 10 and 4-fold, respectively, within 16 h of incubation versus 4 h cultures (Figure 2C,D), while little changes were detected in the levels of benzyl and isoamyl alcohols. It is reported that the aforementioned alcohols including phenylethyl, benzyl and isoamyl alcohols are hyphae-inhibiting metabolites [3,12,23], while tyrosol is a filamentation and biofilm-forming metabolite [16]. The production of phenylethyl, benzyl and isoamyl alcohols is consistent with the yeast form of *C. auris.*

**Figure 5:**
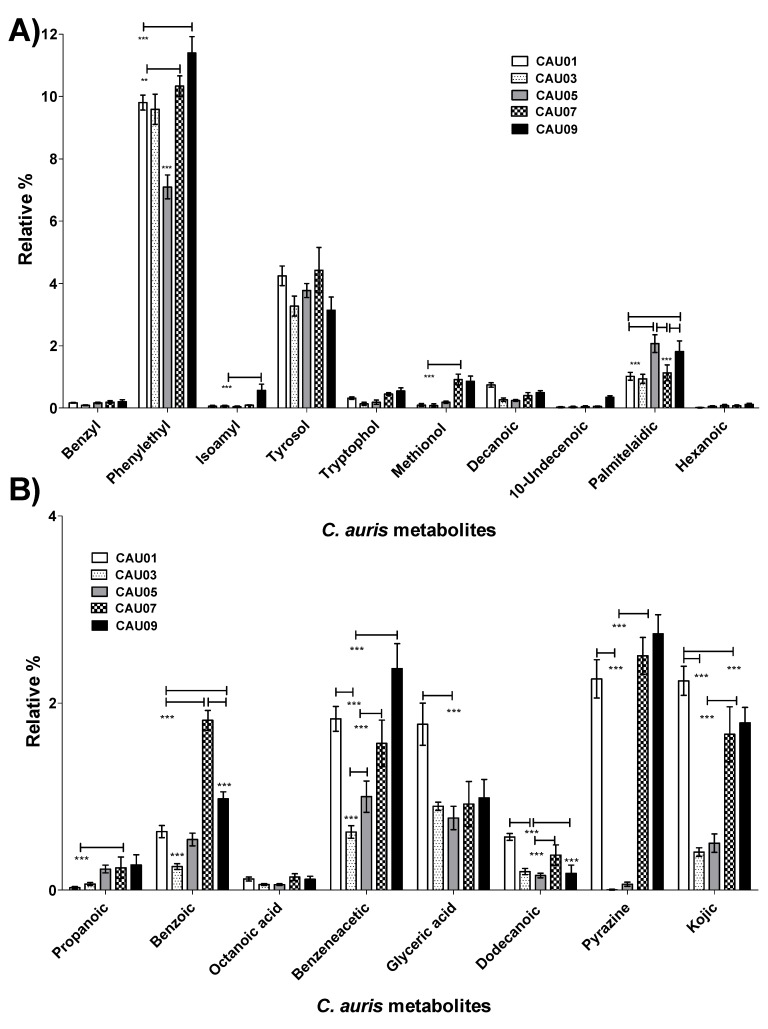
Comparison of relative percentages of metabolites produced by different *C. auris* strains. (**A**) Proposed metabolites employed in morphogenic changes. (**B**) Proposed metabolites produced by the organism for the purpose of protection and colonization. The quantity of each metabolite was represented as the relative percentage by measuring the area under the peak of each metabolite in relation to the total areas of all other metabolites detected in the extract. The data display the mean of the relative percentage ± standard error of the mean. The data was analyzed by two-way analysis of variance (ANOVA). *P* value < 0.05 was considered as significant. The standard error represents the mean of 4 experimental replicates of two independent biological experiments. The level of significance was indicated by asterisks.

The aforementioned changes in metabolites were compared among four other *C. auris* strains along with *C. albicans* ATCC10231 (Heatmap in Figure 3, Figure 5A and Supplementary Table S1). A similar pattern was obtained in all other *C. auris* strains. Specifically, *C. auris* strains secreted high percentages of aromatic alcohols such as phenylethyl alcohol (~7–11%), benzyl alcohol (~0.1–0.2%), and at a lower extent, isoamyl alcohol. Palmitelaidic acid (~1–2%) was also detected in *C. auris* strains. In contrast, none of these metabolites was identified in *C. albicans* ATCC10231 except decanoic acid which was detected in very low amount (0.09 ± 0.023) (Figure 5A and Supplementary Table S1). These results indicate that both phenylethyl alcohol and tyrosol and to some extent palmitelaidic acid are likely critical *C. auris* morphogenic metabolites at the tested condition.

Farnesol was absent in both *C. auris* and *C. albicans* cultures. The absence of farnesol in *C. albicans* culture is expected, since it is a yeast-to-hyphal inhibitory metabolite [15], and the growth condition favored hyphal growth. It is prudent to mention that *C. auris* harbors a homologue for *C. albicans* farnesyl synthase, the rate-limiting enzyme in farnesol biosynthesis [26], with 79% amino acid identity. Equally important, non-*albicans* species of *Candida* are known to produce ~8–35 times lower levels of farnesol when compared to *C. albicans* [27]. Therefore, the lack of detection of any farnesol could be due to lower limits of production consistent with what is seen with non-*albicans* species. This result indicated that farnesol expression was not required in *C. auris* culture condition under study and hence confirmed that other factors may be involved in maintaining the growth of *C. auris* as yeast form. Importantly, farnesol is known to have reversal effect on tyrosol [16]; and production of tyrosol by *C. auris* was favored at this condition, a condition suitable for biofilm development. Collectively, these results indicate that fundamental biological processes are under complex positive and negative control by environmental conditions in which these aromatic alcohols are secreted. Future studies focusing on gene expression analysis and investigation of transcription factors that govern yeast-to-hyphae switch might shed light on the reasons why *C. auris* lacks the ability to form true hyphae.

Decanoic, 10-undecenoic and palmitelaidic were acid metabolites identified in *C. auris* cultures that have never been detected by other *Candida Spp.* (Figure 3, Figure 5A and Supplementary Table S1). This result raises the possibility of using these acid metabolites as biomarkers to aid in the organism diagnosis. The detected metabolites were also reported as hyphae-inhibitory substances [28,29,30], a situation may favor the growth of the organism in the yeast form.

### 2.2. C. auris Produced Auto-Protective/Auto-Toxic Metabolites

Some microbial metabolites exert toxic effects on host cells and/or modulate host immunity in order to facilitate microbial invasion [31]. *C. auris* secreted fatty acids such as capric, undecylenic and palmitelaidic acids (Figure 3, Figure 4A and Figure 5A), known to impair the ability of host natural response to eliminate microorganisms [32]. *C. auris* also secreted propanoic acid that can exert immune-suppressive activity [33] and pyrazine derivative that may help in host-microbe colonization [34] (Figure 4B and Figure 5B). Furthermore, *C. auris* produced autotoxic metabolites including tryptophol [35], propanoic [36], benzoic [37], octanoic acid (caprylic acid) [38], benzeneacetic acid (phenyl acetic acid) [37], glyceric [39], kojic [40,41], dodecanoic (lauric) [42,43] acids and pyrrolo[1,2-a]pyrazine-1,4-dione, hexahydro-3-(2-methylpropyl) [44] that can facilitate its own growth and hence access more nutrients (Figure 4B and Figure 5B). Although the production of the aforementioned metabolites was differ between different *C. auris* strains, pyrazine derivative, benzeneacetic acid, kojic acid, glyceric acid and benzoic acid were produced in higher levels when compared to other auto-protective metabolites (Figure 4B and Figure 5B), which may indicate their favored production by the organism. On the other hand, out of the aforementioned auto-protective metabolites, *C. albicans* showed production of very high level of propanoic acid (~11.35 ± 0.24) (Figure 3 and Supplementary Table S1). Autotoxic/auto-protective metabolites are known to increase nutrient availability and hence allow better colonization of the pathogenic organism in addition to its immune-suppressive effects [35].

### 2.3. C. auris Produced Metabolic Fermentation Products, Known for Colonization and Invasion

Within the host, the pathogen must efficiently compete for nutrients with host cells [45] and its metabolism is affected in a niche-specific fashion [46]. Microbial pathogens produce metabolic products to overcome the host resistance mechanisms and hence allow the pathogen to colonize and invade. Such metabolic fermentation products may modulate the host immune system and change the body temperature or pH [47,48]. Compared to *C. albicans*, *C. auris* produced fermentation metabolic products including hexanoic acid (caproic acid), 2,3-butanediol [49], 2-propenoic acid (acrylic acid) [50], 3-hydroxypropanoic acid (hydracrylic acid) [51] and the yeast-specific fermentation product methionol [52] (Figure 3 and Supplementary Table S1).

## 3. Materials and Methods

### 3.1. Organisms and Culture Conditions

Five different *C. auris* clinical isolates were obtained from Centers for Disease Control and Prevention (CDC, Atlanta, GA, USA). These were CAU-01 (East Asian clade, ear), CAU-03 (African clade, blood), CAU-05 (South American clade, blood), CAU-07 (South Asian clade, blood), and CAU-09 (South Asian clade, bronchoalveolar lavage [BAL]). *C. albicans* reference strain ATCC10231 was used as a control. *Candida* isolates were allowed to grow separately in yeast nitrogen base (YNP) supplemented with 2% glucose at 37 °C for 16 h. Cells were washed with phosphate buffered saline (PBS), and the inoculums adjusted to 5 × 10^6^ yeast/mL with RPMI-1640 (Sigma-Aldrich, Torrance, CA, USA) using hemacytometer [53]. The cultures were further incubated for 24 h at 37 °C in 125 mL Corning culture flasks, after which cell-free supernatants were collected by filtration using 0.2 μM Whatman filter paper.

### 3.2. Preparation of Samples for GC-MS Analysis

The filtered supernatants collected separately from each *Candida* isolate was extracted with chloroform (Fisher Scientific, Santa Clara, CA, USA). The chloroform layer was dehydrated over anhydrous sodium sulphate (Fisher Scientific) followed by evaporation using rotatory evaporator (Buchi, Essen, Germany). The residue collected from each extracted culture was dissolved in 500 µL chloroform prior to GC-MS injection. Furthermore, 100 µL of the chloroform extract was derivatized by adding 50 µL of *N*-trimethylsilyl-*N*-methyl trifluoroacetamide and trimethylchlorosilane (MSTFA + 1% TMS) followed by incubation at 50 °C for 30 min prior to GC-MS analysis.

### 3.3. GC-MS Spectrometry

GC–MS analysis was performed using a QP2010 gas chromatography-mass spectrometer (GC-2010 coupled with a GC–MS QP-2010 Ultra) equipped with an auto-sampler (AOC-20i+s) from Shimadzu (Tokyo, Japan), using Rtx-5ms column (30 m length × 0.25 mm inner diameter × 0.25 µm film thickness; Restek, Bellefonte, PA, USA). Helium (99.9% purity) was used as the carrier gas with the column flow rate of 1 mL/min. The column temperature regime was initially adjusted at 35 °C for 2 min; followed by an increase in a rate of 10 °C/min to reach 250 °C. The temperature was then increased by 20 °C/min until reaching 320 °C and kept for 23 min. The injection volume and injection temperature were 1 µL and 250 °C using splitless injection mode, respectively. The mass spectrometer operated in electron compact mode with electron energy of 70 eV. Both the ion source temperature and the interface temperature were set at 240 °C and 250 °C, respectively. The MS mode was set on scan mode starting from 35 to 450 *m*/*z* with a scan speed of 1428. Data collection and analysis were performed using MSD Enhanced Chemstation software (Shimadzu). Product spectra were identified by comparison of the measured fragmentation patterns to those found in the NIST 08 Mass Spectral Library.

### 3.4. Bioinformatics detection of farnesyl synthase protein in C. auris

A local BLAST database of *C. auris* genome was created using Basic Local Alignment Search Tool plus (BLAST+) [54]. Farnesyl synthase protein sequence (Accession #KGQ89011) from *C. albicans* was downloaded from NCBI and used to search the generated *C. auris* genome using tblastn command. The search revealed a sequence with 79% identity within 577 score.

### 3.5. Statistical analysis

The data was collected and graphed using either Excel to generate the heatmap or Graph Pad (5.04, La Jolla, CA, USA) for Windows to generate the Box-and-Whiskers Plots and group comparison. The quantification of metabolites extracts were analyzed by one-way analysis of variance (ANOVA) using Bonferroni’s Multiple Comparison Test or two-way analysis of variance as indicated per each graph. *P* value < 0.05 was considered significant. The standard error represents the mean of 4 replicas of two independent experiments (two experimental replicates per each independent biological experiment). Each extract was divided into two and extracted separately followed by derivatization prior to GC-MS analysis.

## 4. Conclusions

Metabolites produced by microorganisms may be used as communication signals that allow the microorganisms to share information and hence provide some regulatory responses during infection. Metabolic profiling can be of great help to identify critical determinant of pathogens and hence can control disease progression. *Candida spp.* is known for secreting metabolites that control its morphogenesis. *C. auris* is a newly-emergent *Candida* species that is maintained in the yeast phenotype during its growing stages. Analysis of *C. auris* cultures by GC-MS showed that the fungus produces diverse hyphae-inhibiting metabolites in addition to biofilm-forming tyrosol that are distinct from *C. albicans*. The results provided in this research is the first to identify *C. auris* metabolic profiling; and thus can shed light on the virulence of this multi-drug resistant yeast and may eventually lead to the development of new strategies for in deep investigation including gene expression analysis and study of morphogenesis-regulatory pathways.

## Supplementary Materials

The supplementary materials are available online.
