# Supplementary material for: Metabolic Profiling of Candida auris, a Newly-Emerging Multi-Drug Resistant Candida Species, by GC-MS
Source: Molecules. 2019 Jan 22;24(3):399. doi: 10.3390/molecules24030399 (PMC6384714; doi:10.3390/molecules24030399)
Supplement: Supplementary file 1 [file molecules-24-00399-s001.pdf]

Supplementary Table

Metabolic Profiling of *Candida auris,* a Newly-Emerging Multi-Drug Resistant *Candida* Species, by GC-MS

Supplementary Table 1. Metabolites produced by *C. auris* liquid cultures compared to *C. albicans* culture at hyphae growing conditions. The quantity of each metabolite was represented as a relative percentage by measuring the area under the peak of each metabolite in relation to the total areas of all other metabolites detected in the extract. The data is presented as mean ± standard error of four replicas.

Metabolites	*Candida* strains					
	CAU01	*CAU03*	*CA05*	*CAU07*	*CAU09*	*C. albicans*
**Benzyl**	0.17 ± 0.013	0.093 ± 0.015	0.17 ± 0.022	0.19 ± 0.05	0.2 ± 0.06	0
**Phenylethyl**	9.80 ± 0.24	9.59 ± 0.48	7.097 ± 0.4	10.33 ± 0.32	11.39 ± 0.5	0
**Isoamyl**	0.057 ± 0.034	0.068 ± 0.023	0.043 ± 0.03	0.097 ± 0.006	0.57 ± 0.19	0
**Tyrosol**	4.245 ± 0.3	3.27 ± 0.32	3.77 ± 0.22	4.425 ± 0.7	3.137 ± 0.4	0
**Tryptophol**	0.32 ± 0.04	0.14 ± 0.05	0.19 ± 0.068	0.452 ± 0.04	0.55 ± 0.1	0
**Methionol**	0.098 ± 0.05	0.082 ± 0.05	0.19 ± 0.039	0.917 ± 0.17	0.86 ± 0.16	0
**Decanoic**	0.74 ± 0.073	0.267 ± 0.06	0.247 ± 0.028	0.402 ± 0.09	0.5 ± 0.06	0.09 ± 0.023
**10-Undecenoic**	0.028 ± 0.017	0.034 ± 0.02	0.046 ± 0.026	0.05 ± 0.02	0.34 ± 0.05	0
**Palmitelaidic**	1.02 ± 0.12	0.939 ± 0.14	2.072 ± 0.28	1.132 ± 0.25	1.8 ± 0.34	0
**Hexanoic**	0.015 ± 0.009	0.054 ± 0.03	0.082 ± 0.04	0.075 ± 0.03	0.12 ± 0.03	0
**Propanoic**	0.025 ± 0.013	0.066 ± 0.02	0.225 ± 0.04	0.24 ± 0.11	0.27 ± 0.11	11.35 ± 0.24
**Benzoic**	0.627 ± 0.065	0.252 ± 0.03	0.542 ± 0.07	1.8175 ± 0.1	0.977 ± 0.07	0
**Octanoic acid**	0.119 ± 0.021	0.060 ± 0.01	0.059 ± 0.009	0.14 ± 0.04	0.117 ± 0.03	0
**Benzeneacetic**	1.83 ± 0.13	0.622 ± 0.067	1.0 ± 0.16	1.57 ± 0.24	2.37 ± 0.3	0
**Glyceric acid**	1.77 ± 0.22	0.897 ± 0.043	0.77 ± 0.12	0.92 ± 0.2	0.987 ± 0.19	0
**Dodecanoic**	0.57 ± 0.036	0.198 ± 0.034	0.157 ± 0.03	0.375 ± 0.1	0.18 ± 0.08	0
**Pyrazine**	2.26 ± 0.2	0.005 ± 0.005	0.063 ± 0.022	2.507 ± 0.19	2.742 ± 0.2	0
**Kojic**	2.24 ± 0.15	0.407 ± 0.05	0.502 ± 0.098	1.667 ± 0.3	1.79 ± 0.16	0
